# Management of two variants of the OEIS complex (Omphalocele, Exstrophy, Imperforate anus, Spinal defects) : case reports and literature review"

**DOI:** 10.1186/s12887-026-06775-w

**Published:** 2026-04-17

**Authors:** Hebatallah Taher, Burhansyah Azhar, Nadeen Alasbahi, Sally Emad Eldin, Khaled Salah, Belal M. Alezaby, Ahmad E. Fares, Mennatullah Alaa Shehab, Mostafa Gad

**Affiliations:** 1 Department of pediatric surgery, Cairo University Children Hospital, Cairo, Egypt; 2 Cairo University Teaching Hospital, Cairo, Egypt; 3 Department of pediatric radiology, Cairo University Children Hospital, Cairo, Egypt; 4 Department of pediatric surgery, Fayoum University, Fayoum, Egypt; 5 Cairo University, Cairo, Egypt

**Keywords:** Anorectal Malformation, Ectopic anus - caudal duplication syndrome, Perineal Lipoma, Clitoral duplication

## Abstract

**Background:**

There is a wide spectrum of anorectal malformations (ARMs). The majority of ARM types can be diagnosed thorough perineal examination and radiological investigations such as MRI and distal loopogram. The most important aim of ARM repair is keeping the rectum in the muscle complex to protect continence, but still there are important factors that influence continence such as spine anomalies and associated urological anomalies.

**Case presentation:**

In our report we describe two patients with ARM and abnormal anal sphincteric locations. The first case was a two-year-old female patient who presented with a massive perineal mass and peculiar female external genitalia that covers the entire perineum. Examination under general anaesthesia (EUA) revealed an extensive perineal lipoma that extends from the posterior point of the coccyx to the middle anterior separated pubic bone overlying a cavity separating it from a normal vaginal introitus containing normal urethral meatus, bicuspid Hymen and anorectal malformation with a vestibular fistula. MRI pelvis showed a large perineal lipoma. Surgical removal of the perineal mass, restoration of the perineal body and external genitalia, and anorectoplasty were performed. The second case was a 13-year-old male who is known to have bladder exstrophy but presented with mucosal bridges producing mucous and abnormally located anus. Excision of the mucosal bridge and confirming the accurate location of the anus within the muscle complex was performed.

**Conclusions:**

It is important to look for and put into consideration other anomalies which can be associated with ARMs and influence the continence, in addition, to accurately locating the sphincter muscle complex using muscle stimulation to ensure placement of the anus within the complex to provide the best bowel function outcome for these cases.

**Supplementary Information:**

The online version contains supplementary material available at 10.1186/s12887-026-06775-w.

## Background

**Fig. 1:**
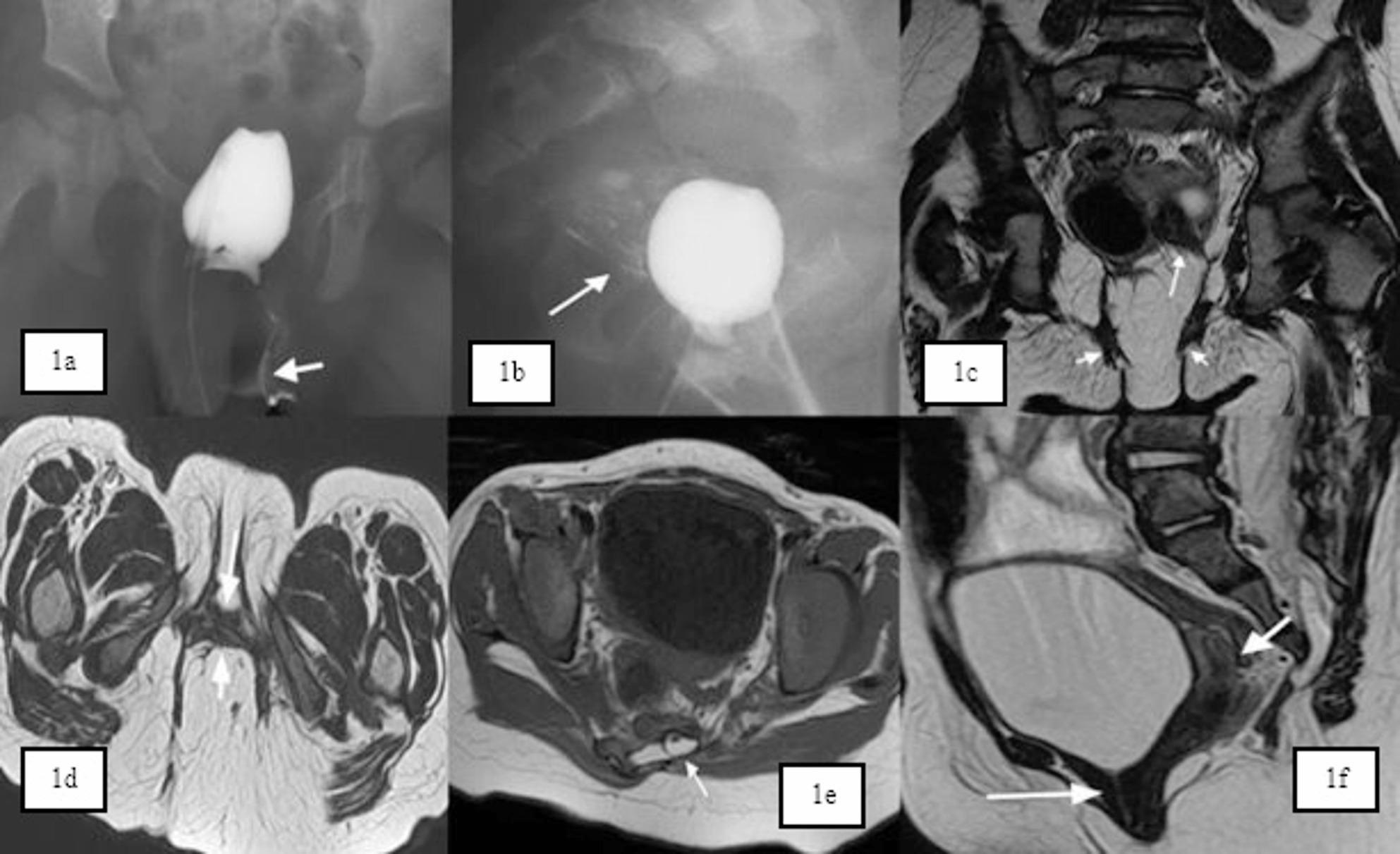
**a** & **b**: Genitography examination and opacification of vagina and uterus. **c - f**: MRI pelvis

There is a wide spectrum of anorectal malformations (ARMs). The majority of ARM types can be diagnosed thorough perineal examination and radiological investigations such as MRI( Fig. 1a and 1f) and distal loopogram. The most important aim of ARM repair is keeping the rectum in the muscle complex to protect continence, but still there are very important factors that influence continence such as spine anomalies such as sacral ratio, tethered cord and associated urological anomalies. Those factors should be put into consideration during patient follow up and quality of life assessment. In this report, we describe two patients with ARM who presented with abnormal location of the anal sphincter.

### Case report

#### Case 1

**Fig. 2:**
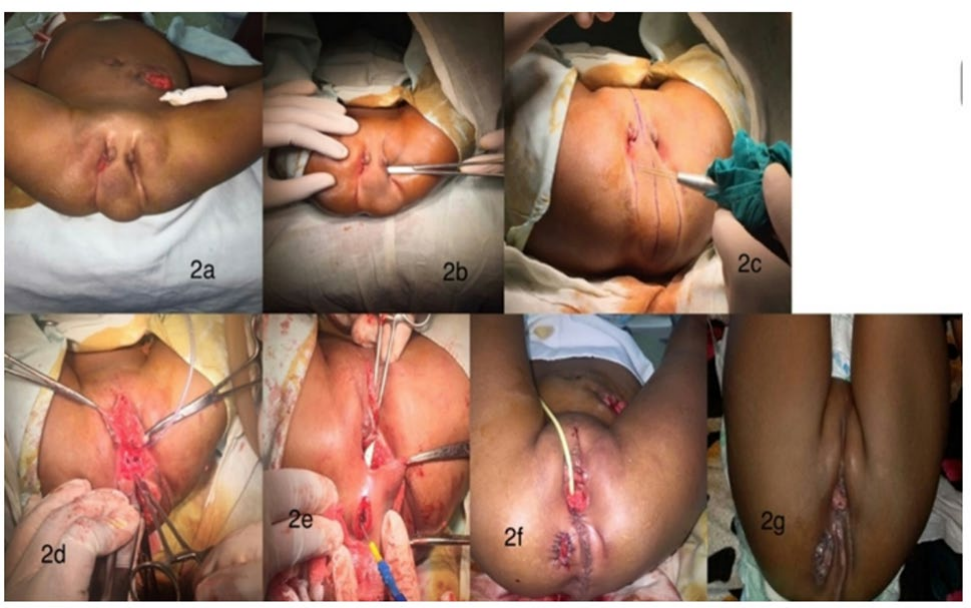
**a** & **b**: preoperative appearance of pernieum, **c** -**f**: operative steps of the operation. **g**: postoperative appearance few weeks later

A two-year-old female child was referred to our paediatric surgery tertiary centre to surgically manage her ARM after having a loop sigmoid colostomy fashioned locally at the age of 2 days. The child was thriving normally with no gross developmental delay. Upon examining her perineum, there was absent anal orifice with a large perineal soft mass, which was not well circumscribed, extending from the posterior part, where the coccyx is, to anteriorly at a mildly separated pubic bone covering the normal anal position and the female external genitalia. There were two well-developed clitorises with no visible vaginal introitus or urethra opening. There were two well-developed labia majora but widely separated by the perineal mass (Fig. 2a and 2g).

Radiological tests were requested, and the consent was given by the parents to perform an examination under anaesthesia (EUA) and proceed with corrective surgery.There was no associated renal abnormalities on abdominal US. Genitography and distal loopogram were performed using a catheter inserted through the right introital orifice, showed opacification of the uterine cavity and a vagina (Fig. 1a), there was minimal opacification in the rectum, which indicated a recto-vestibular fistula(Fig. 1b). MRI Pelvis showed normal appearances of vagina, uterus and ovaries for the patient’s age (Fig. 1c-e), It also showed partial sacral agenesis, in which lower sacral segment is para midline oriented, along with skin covered sacral spina bifida, small intraspinal sacral lipoma and tethered cord (Fig. 1f).

EUA of the perineum revealed a perineal mass dividing the single laterally placed labia majora on either side of the introitus duplication, which had two orifices (Fig. 2a). There was a duplicate of the clitoris visible from both sides. As a catheter was inserted through one orifice and left through the other, indicating the two opening on both sides were communicating with each other (Fig. 2b). The orifices of the vagina and urethra were not detectable externally, but intermittent urinary voiding from each opening of the introitus was seen. The anal orifice was not present.

Using muscle stimulator, the muscle complex was detected to the right side of the midline where the point of maximal contraction was visible (Fig. 2c).

Decision was to proceed with corrective surgery in the form of excision of the accessory clitoris and perineal mass with same-setting anoplasty. Perineal examination by muscle stimulator to mark the location for neo-anus based on the point of maximum contraction of muscle complex. There were very minimal contractions on each side of the swelling which was more prominent on the right side.

Then, midline skin incision was performed from the symphysis pubis anteriorly till the tip of coccyx posteriorly to avoid injury of important structures. The mass was dissected from both sides followed by transverse incision into the mass in a secure plane, over an instrument introduced from the original perineal opening to the other one to elevate the soft tissue mass away from the underlying structures, creating an upper and a lower soft tissue flaps (Fig. 2d). Under the soft tissue mass, there was a normal vaginal introitus with a thin bicuspid hymen and a normal external urethral orifice, urinary catheter 8 Fr Silicon was inserted (Fig. 2d). The lower soft tissue mass was then dissected from its posterior attachment under vision, after elevation of the mass from its bed a tiny orifice of a tubular structure was found at the base of the Hymen, a Nelaton catheter was introduced through this orifice which proceeded till it came out from the colostomy confirming the presence ofa vestibular fistula. Dissection commenced inferiorly till normal perineal tissue reached. Multiple stay sutures to the rectal fistula were placed and dissection of the rectum all around by a needle diathermy was performed. the common wall between rectum and vagina was separated uneventfully with a good length of rectal stump to reach the previously marked anus site. Then, the upper half of the mass along with the left clitoris was excised with preservation of the right clitorus because it was closer to the midline.

Finally, the muscle complex was assessed again using the muscle stimulator, it was still shifted to the right side but weak contractions. Decision to proceed with a small vertical incision in the middle of the muscle complex which was deepened by blunt dissection (Fig. 2e) followed by a pullthrough of the rectum and stitching it to the middle of the muscle complex, anoplasty was done by vicryl 5/0 sutures (Fig. 2f).

The histopathology analysis of the excised mass showed mature fat cells, confirming the diagnosis of a lipoma. Anal dilatation using Hegar dilator size 12 was started two weeks post-operatively and planned to increase size of the dilator gradually till we reach the appropriate size for age (Fig. 2g).

#### Case 2

A Thirteen-year-old male with Ectopia vesica presented to the unit with discharge from mucosal bridge area near the anus which was causing distress for him. He also had an abnormally sited anus and family were querying if the location of this anus was normal. The patient had a sigmoid colostomy in-situ. On examination, the patient had a neglected bladder exstrophy, mucosal bridge in the midline and an anus in the right gluteal area away from midline. After patient and family counselling, they reached a conclusion that they are keen to get rid of this mucous producing mucosal bridge as well as confirm if the anus is in a normal location.

EUA of the perineum using muscle stimulator revealed that muscle complex was shifted to the right side and the anus is in the centre of the muscle complex. There were was no contractions in the midline. (Supplementary video 1) a dentate line was seen upon examination.

**Fig. 3:**
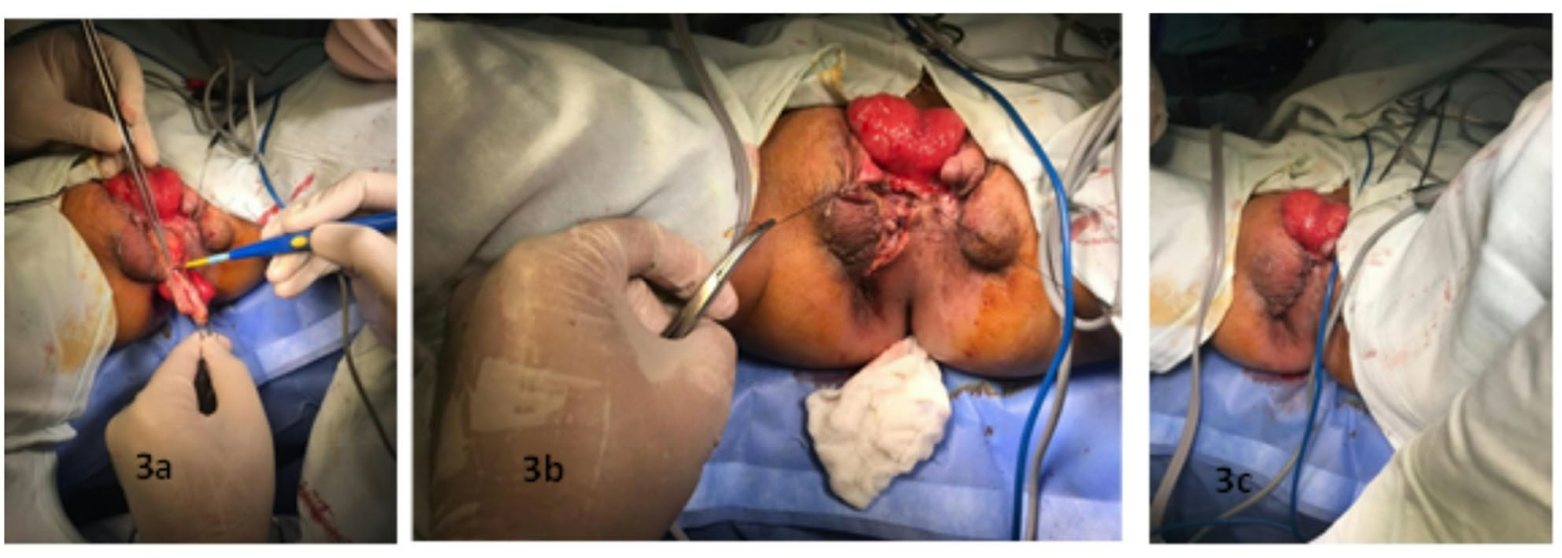
**a** : The excision of mucosal bridges. **b** : Skin were sutured and closed. **c** : Immidiate post operative picture

We diagnosed it as ectopic and and decided to leave the anus in the ectopic location and to proceed with excision of the mucosal bridge (Fig. 3a) and closure of skin to create a skin bridge between the bladder exstrophy and the anus the skin was closed (Fig. 3b). The patient was then scheduled for definitive repair of his bladder exstrophy with the urology and orthpaedic teams. (Fig. 3c)

The bowel function in first patient is poor and is scheduled for antegrade continence enema(ACE) and second patient still has his stoma until urological procedures are carried out .

## Discussion

Placing the neorectum in muscle complex is an important step of the operation to maintain normal continence, surgeons are familiar with placing the neorectum in the midline after confirming with muscle stimulation, however occasionally as in our report the muscle complex can be abnormally positioned and therefore the neo anus is not necessarily positioned in midline as in our patients.

Our first patient had recto-vestibular fistula, which is considered to be the most common ARM defect in females [1]. Although in general the malformation considered as “good side” of the spectrum of anorectal defects, the study conducted by Peña et al. showed 35% of cases were associated with tethered cord [1]. which is higher than the average of other anorectal malformations. Peña assumed that it can be explained by the high incidence of presacral masses in this type of malformation, which also at the same time tethered cord is very common in the cases with presacral masses [1].

Our first patient had a tethered cord; however, the association was not a presacral mass but rather intra spinal sacral and a perineal lipoma elongated from tip of coccyx to the anterior part of the diastatic pubic bone. A systematic review of the literature was conducted to search for similar cases and despite the few reports on ARM associated with different perineal masses including lipoma together with accessory labioscrotal folds in females and scrotum in males, perineal Lipoma is a rare association with ARM, Peña reported two cases [2] which were included in his study of 22 patients who had ARM associated with perineal masses excluding presacral masses, excluding patients with lipomas of spinal cord, sacral dimples, presacral teratomas, and any mass located elsewhere in pelvis. Surgical approach [2] of the perineal lesions is relatively simple, but if associated with anorectal malformation with the lesion situated in the future anal place, then techically surgery will be so challenging. Peña et al. believe, that most of them should be excised carefully, preserving the muscle complex and future continence.

Wester and risto Rintala [3], reported 6 patients with ARM and perineal lipomas of which 2 female patients had rectovestibular fistula and lipoma, also stated that such lesions impact surgical management but while in Peña’s series these small lesions are not affecting the bowel control, wester and Rintal had one patient who had unfavourable bowel control and concluded that the lesion, including lipoma, may interfere and give unsatisfactory result. They explained [3] that in majory of their patients there is a distortion of the sphincter, and this is an essential factor to determine the last functional result. They recommend that the parents should be informed about the association of perineal lipomas and anorectal malformation as it will have negative functional outcome. They also stressed [3] on the importance to histologically examining the excised perineal lipomas to exclude lipoblastoma, which is rare tumour consisting of lipoblast [4, 5]. These tumors are benign, but they may grow rapidly even McVay et al. [3] reported recurrence of lipoblastoma in 12.5% of the cases.

Peña et al. [2] also stressed on the importance of accurate pathological examination of the excised specimens. According to a recent review there were 50 cases in English literature report congenital perineal lipoma, including perianal lesion [6]. There are variations as regard the size and location among the patients, therefore the surgical strategy may differ according to these association. They involved [6] 25 males and 25 females, which 74% of them (37 cases) having other anomalies, in which mostly ARM (40%), then followed by labio scrotal fold or accessory scrotum in 28% of them (14 patients) four of which had 2 accessory labioscrotal fold and 2 had vestibular fistula and an accessory labioscrotal fold. Other anomalies included urogenital malformation, disorder of sexual differentiation, and congenital pulmonary airway malformation.

The triad that consisting of anorectal malformation (ARM ) perineal mass, and labioscrotal fold malformation has rarely reported before. Kai Wang et al. [7] recently (2019) reported a series of 7 patients 6 females and one male The ARM type was all rectoperineal fistula. Other pathologies included Lipoma (three cases), fibroma (one case), lipofibroma (one case), angiolipoma (one case), and mesenchymal hamartoma (one case). All of them had satisfactory bowel control in the follow up period. They suggested that ARM, along with its association of Perineal mass and Labioscrotal fold malformation have a low incidence, are infrequently seen in clinical practice, and may be more common in females. The triad [7] is naturally consisting of abnormal excessive growth of the intervening mesenchymal tissue, and the continuation of caudal development of normal labioscrotal fold will be distrupted as well as affection of the extension of urorectal septum and occurrence of ARM with the type of ARM determining the future prognosis.

Both cases are moderate and minor variants of the OEIS complex/syndrome (Omphalocele, Exstrophy, Imperforate anus, Spinal defects) in which the spectrum of combination of exstrophy, imperforate anus, and spinal defects is seen encompassing abdominal wall, urological, genital, gastrointestinal, spinal and musculoskeletal defects in various combinations and permutations [8, 9].

The first case of two year old girl had covered bladder exstrophy [10] (This refers to a variation where the bladder is partially or completely covered by skin, but still has subtle pubic symphysis separation skeletal abnormalities), giant perineal lipoma, two well-developed clitorises, the vaginal introitus duplication with bicuspid hymen, recto-vestibular fistula with anorectal malformation and ectopic anal sphincter complex and partial sacral agenesis along with skin covered sacral spina bifida, small intraspinal sacral lipoma and tethered cord. The second patient had neglected classic bladder exstrophy (the bladder is open and exposed on the lower abdomen, with the bladder’s inner surface fused to the abdominal skin with or without epispadias), perineal groove [11] in which the roof of anovestibular or rectovestibular fistula remains and the floor disappears. there are three anterior extensions from the abnormal anal canal in anorectal malformations-Anovestibular fistula, perineal canal and a perineal groove-(which is an abnormal mucosa lined bridge, groove or indentation in the perineal area), anorectal malformation with split or duplicated anal canal or proctodeum and ectopic sphincter muscle complex.

The anatomy of caudal duplication involves a spectrum of presentations. This is not the first report of a clitoral duplications [12] our first patient has complete duplication of the clitoris for which excision is only treatment required [13, 14]. There should be clear differentiation between clitoral duplication [15] and bifid clitoris. It is usually associated with bladder exstrophy, with urethral duplication, or with epispadias. This condition is uncommon, and the incidence counted one among 480,000 females.

For the first patient long term following up includes bowel management program due to increased likely hood of continence affection as result of vertebral anomalies, as for the second patient urological follow up with urology team is of utmost importance.

Given the complex nature of those anomalies, it would be of benefit to adopt recent advances in technology and artificial intelligence to gather information about those complex malformations to train artificial intelligence models to help in diagnosing and managing such conditions [15–21].

## Conclusion

It is important to look for and put into consideration other anomalies, such as spine anomalies, perineal masses, presacral masses and urological anomalies, which can be associated with ARMs and influence the continence, in addition, to accurately locating the sphincter muscle complex using muscle stimulation to ensure placement of the anus within the complex to provide the best bowel function outcome for these cases.

## Supplementary Information

Supplementary Material 1.

## Data Availability

Yes, available and provided upon request. For further request please contact this given e-mail : Hebatallah.Taher@kasralainy.edu.eg.
